# Supplementary material for: Maternal knowledge and practice of safe infant sleep position in South Ethiopia: Implications for preventing sleep-related infant deaths
Source: PLoS One. 2026 Feb 3;21(2):e0339408. doi: 10.1371/journal.pone.0339408 (PMC12867215; doi:10.1371/journal.pone.0339408)
Supplement: S1 File — (DOCX) [file pone.0339408.s001.docx]

# **Questionnaire Amharic version**

|  | **ክፍል አንድ፡- አጠቃላይ የግለሰቡ የግል እና የቤተሰብ መረጃ** | | |
| --- | --- | --- | --- |
| 001 | ከልጁ ጋር ያለዎት ግንኙነት ምንድነው? | ሀ. እናት ለ. አባት ሐ. ሌላ |  |
| 002 | ዕድሜሽ ስንት ነው? (በአመታት) |  |  |
| 003 | የጋብቻ ሁኔታ | ሀ. ያላገባ ለ. ያገባ ሐ. የተፋታች መ. ባል የሞተባት |  |
| 004 | የሚኖሩበት ቦታ/ስፍራ | ሀ/ ገጠር ለ/ ከተማ |  |
| 005 | የእናትየው የትምህርት ደረጃ | ሀ.መደበኛ ት/ት ያልወሰደ  ለ. የመጀመሪያ ደረጃ (ከ1-8)  ሐ. .ሁለተኛ ደረጃ (ከ9-12)  መ. ከፍተኛ/ከሁለተኛ ደረጃ በላይ |  |
| 006 | ሥራ | ሀ. ተቀጣሪ ለ. ስራ የለለዉ ሐ. በራስ ተቀጣሪ  መ. ተማሪ ሰ. የቤት እመቤት |  |
| 007 | የሕፃኑ ዕድሜ ስንት ነው(በወራት) |  |  |
| 008 | የበተሰብ ብዛት ስንት ነዉ? | ሀ. 1-4  ለ. 5 -8  ሐ. 9 ና በላይ |  |
| 009 | ይህ የመጀመሪያ ልጅሽ ነው? | ሀ. አዎ ለ. አይ |  |

|  | **ከእርግዝና ጋር ተያያዥ መረጃዎች** | | |
| --- | --- | --- | --- |
| 010 | ለስንት ጊዜ ያህል አርግዝሻል? |  |  |
| 011 | ስንት ልጅ ወለድሽ? |  |  |
| 012 | የመጨረሻው እርግዝና ቅድመ ወልድ ክትትል አድርገሻል? | ሀ. አዎ ለ. አይ |  |
| 013 | ስንትጊዜ እርግዝና ክትትል አድርገሻል? |  |  |
| 014 | የት ነው የወለድሽው? | ሀ. ቤት ለ. በጤና ተቋማት |  |
| 015 | የልጅሽ ፆታ | ሀ. ወንድ ለ. ሴት |  |
| 016 | ስንት ልጆች አሉሽ? |  |  |
|  | የእውቀት እና የተግባር ጥያቄዎች | | |
| 017 | ስለድንገተኛ የጨቅላ ሕጻናት ሞት ሰምተው ያውቃሉ? | ሀ. አዎ ለ. አይደለም | ለ |
| 018 | በእንቅልፍ ጊዜ ሕፃን የሚተኛበት የመኝታ አተኛኘት ሁኔታ ሰምተሻል? | ሀ. አዎ ለ. አይ |  |
| 019 | ለቀደመው ጥያቄ መልሱ አዎ ከሆነ ከየት ነው የሰሙት? | ሀ. አያት ለ. የጤና ባለሙያዎች  ሐ. ጓደኞች |  |
| 020 | የማንን ምክር ተግባራዊ ማድረግ ይፈልጋሉ? | ሀ. የሴት አያቶችን ለ. የጤና ባለሙያዎች ሐ. ጓደኞች |  |
| 021 | አራስ ሕፃናትን እንቅልፍ ለማስተኛት ምን ይመረጣል? | ሀ. ጎን ለ. ጀርባ  ሐ. ሆዱ መ. አላውቅም |  |

| 022 | ሕፃን ለመተኛት ተስማሚ አካባቢ የት ነው? | ሀ. ከወላጆች የተለየ ክፍል።  ለ. ከወላጆች ጋር ተመሳሳይ ክፍል  ሐ. አላውቅም |  |
| --- | --- | --- | --- |
| 023 | ለህጻናት የሚተኛበት ተስማሚ ቦታ | ሀ.የተለየ አልጋ  ለ. ከወላጆች ጋር ተመሳሳይ አልጋ  ሐ. አላውቅም |  |
| 024 | ከፍራሽ ስር ትራስ ለጨቅላ ህጻናት ይመረጣል | A. አዎ ለ. አይ |  |
| 025 | አስተማማኝ የእንቅልፍ አስተኛኜት ድንገተኛ የጨቅላ ሕጻናት ሞት ይከላከላል | ሀ. አዎ ለ. አይ |  |
| 026 | ብቸኛ ጡት ማጥባት ማለት | ሀ. የጡት ወተት ለስድስት ወራት ብቻ መመገብ ማለት ነው።  ለ. የጡት ወተት እና ተጨማሪ ምግብን በማጣመር መመገብ ማለት ነዉ። |  |
| 027 | ልጅዎ በግል በአልጋ ላይ ይተኛል? | ሀ. አዎ ለ.አይ |  |
| 028 | ጨቅላዎ ከእርስዎ ወይም ከአሳዳጊ በተለየ ክፍል ውስጥ ይተኛል? | አ. አዎ ለ. አይ |  |
| 029 | ሕፃኑ አልጋውን ከአሳዳጊው ጋር ይጋራል? | ሀ. አዎ ለ. አይ |  |
| 030 | በእርግዝና ወቅት እና ከተወለደ በኋላ ለጢስ/ለስጋራ መጋለጥ አለብዎት | ሀ. አዎ ለ. አይ |  |
| 031 | የእርስዎ ጨቅላ ከጀርባ ውጪ እንቅልፍ ይተኛል | ሀ. አዎ ለ. አይ |  |
| 032 | ህፃኑ አብዘኛውን ጊዜ እንዴት ይተኛል? | ሀ. ሆዱ ለ. ጀርባ ሐ. ጎን |  |
