# Supplementary material for: Maternal knowledge and practice of safe infant sleep position in South Ethiopia: Implications for preventing sleep-related infant deaths
Source: PLoS One. 2026 Feb 3;21(2):e0339408. doi: 10.1371/journal.pone.0339408 (PMC12867215; doi:10.1371/journal.pone.0339408)
Supplement: S2 File — (DOCX) [file pone.0339408.s002.docx]

# **Questionnaire** **English version**

|  | Sociodemographic characteristics |  |  |
| --- | --- | --- | --- |
| 001 | What is your relationship to the child: | 1. Mother 2. Father 3. Other |  |
| 002 | Mother’s age? (In years) | A. Age 18 and under  B.19-24  C.25-30  D.31-36  E.37 and older |  |
| 003 | Where is your current residency? | A. In urban area (City)  B. In rural area |  |
| 004 | What is your current marital status? | Single  Married  Divorced  Widowed |  |
| 005 | Educational status of mother | A. No formal education  B. primary education  C. secondary education  D. higher/above secondary |  |
| 006 | Maternal occupation | A. employed  B. unemployed  C. Self employed  D. Student  E, House wife |  |
|  |  |  |  |
| 007 | What is the household’s monthly income? | A. Less than 1500  B .1501 - 3500  C .3500 above |  |
| 008 | What is the total number of people in this family? | A.1-4  B.5-8  C.9 and above |  |
| 009 | What is the age of the infant (in months)? |  |  |
| 010 | Is this your first child? | A. Yes  B. No |  |

|  | Obstetric factors |  |  |
| --- | --- | --- | --- |
| 010 | How many times did you get pregnant? | A. Primi-gravida  B. Multigravida |  |
| 011 | How many times did you gave birth? | A. Primi-para  B. Multi para  C. Grand multipara |  |
| 012 | ANC follow up during  last pregnancy | A. Yes  B. No |  |
| 013 | How many ANC visit? | A. One visit  B.2-4 visits  C.5 and more |  |
| 014 | Place of birth for recent pregnancy | A. Home  B. At health institutions |  |
| 015 | Sex of your baby | A. Male  B. Female |  |
| 016 | How many children do you have? | A. 1-4  B.5-8  C.9 and above |  |

|  | Knowledge and practice questions |  |  |
| --- | --- | --- | --- |
| 017 | Have you ever heard about Sudden Infantile Death Syndrome (SIDS)? | A. Yes B. No |  |
| 018 | Have you heard about infant sleep position | A. yes  B. no |  |
| 019 | where did you hear about sleep position? | A. My mother  B. mother-in-law  C. grandmothers  D. I didn’t hear |  |
| 020 | Advice from whom you want to apply | A. Grandmothers  B. Health professionals  C. Friends |  |
| 021 | What is preferred sleep position for infants? | A. side  B. back  C. prone  D. I don’t know |  |
| 022 | What is appropriate environment to sleep an infant? | A. Separate room from parents.  B. the same room with parents  C.I don’t know |  |
| 023 | Appropriate place to sleep for infants | A. separate bed/crib  B. The same bed with parents  C.I don’t know |  |
| 024 | Pillow under the mattress preferred for infants | A. yes  B. no |  |
| 025 | SIDS prevented by safe sleep position | A. yes  B. no |  |
| 026 | What do think exclusive breast feeding mean | A. feeding only breast milk for six months  B. Feeding breast milk and complimentary food in combination |  |
| 027 | Is your infant sleep on crib/cot | A. Yes  B.no |  |
| 028 | Does your infant sleep in a separate room from you or a caregiver? | A. Yes  B. Yes, |  |
| 029 | Is an infant Sharing the bed with the caregiver? | A. Yes  B.No |  |
| 030 | Do you have Smoke exposure during pregnancy and after birth | A. yes  B. no |  |
| 031 | IS your infant sleep other than supine | A. Yes  B. No |  |
| 032 | How does the infant usually sleep? | A. Prone  B. Supine  C. Side |  |
