# Supplementary material for: Maternal knowledge and practice of safe infant sleep position in South Ethiopia: Implications for preventing sleep-related infant deaths
Source: PLoS One. 2026 Feb 3;21(2):e0339408. doi: 10.1371/journal.pone.0339408 (PMC12867215; doi:10.1371/journal.pone.0339408)
Supplement: S3 File — (DOCX) [file pone.0339408.s003.docx]

# **Questionnaire wolaytic version**

**Machchanne mayyetuwaa**

Aymalee l” oo? Manta Demissie Tamene Arba Mincce Univurshiyan akkamuwanne payyatetta kollojiyan nattanne nattu payattetta naguwan na” atto diggiriyaa luxxiddi de”agga gidishin qeeri nattu injje ichchisiyo pollowaa ayyettu bollan piliggiddi des. Hegaa gidiyo gishshawu issi layyttappe garssa gididda nattu injje ichchisiyo meeziyaa xelliyaba shishshanawu koyayis. Ha zarottu giddon aybikka nennara gayttiyabi woyko ne suntta xessiyabi xafettenna. Ha piliggetta giddon neeni geliyowode neen ha oyshatta zaranawu immiyo wodeppe attin aybikka nenna qohiyabi deenna. Yattiyo gishawu ha pilgeettan gellanawu mayettaddi?

Ee mayyettas chii mayyetabeykke

ወላይትኛ ቅጂ

|  | Kummetta naqqashaa |  |  |
| --- | --- | --- | --- |
| 001 | Neeni yiirayoo Aybee? | A. Aawaa  B. Aayoo  C. Haraa |  |
| 002 | Layttay neyyoo appunee | A. 18 ppe garssa B. 19- 24  C. 25 – 30 D. 31- 36  E. 37 pee bolla |  |
| 003 | Luxxetta xekkay | A. Xaffiyoganne nababiyoga dandayikke  B. Xafuwaanne nababuwa dandayayis  C. Koyroo xekkaa  D. Na”atto xekkaa  E. xoqqa/Yunvurshiyaa |  |
| 004 | Qatto Awee? | A. Kattamaa  B. Gaxxariyaa |  |
| 005 | Azinaa gelladdi | A.Gellas  B.Gellabikke |  |
| 006 | Ossoy aybee | A. Kawo keetta oso  B. Zal’anchcha  C. Tarka tana aysayis  D. Tamaaree  E. So ossuwaa |  |
| 007 | Kettaa asaa qoday woysee | A.1-4 B.5-8 C.9 nne bolla |  |
| 008 | Ne na”aas/na”eesi woysu aginee? |  |  |
| 009 | Hagee/hanna ne na”ee? | A.Giddes  B.Giddenna |  |

|  | Yelluwaara gayttidabatta |  |  |
| --- | --- | --- | --- |
| 010 | Woysutto sharraddi | A. Koyro  B. Na”anne boll |  |
| 011 | Woysutto yeladdi | A. Issito  B. Issuwappe daro  C. Coraa |  |
| 012 | Sharaa wode payyattetta eqqottan kalladi? | A. Kallaas  B. Kallabikke |  |
| 013 | Appuntto kalladdi? | A. Issitto  B.2-4 gakkanashin  C.5 ppe bolla |  |
| 014 | Awan yelladdi | A. Sooni  B.Payyattetta eqottatun |  |
| 015 | Na”ee/ na”aa qanttay aybee | A. Attuma  B. Macca |  |
| 016 | Niyo Appun natti dii | A. 1-4  B.5-8  C.9 nne coraa |  |

|  | Eraanne polluwaa xelliya oyshshata |  |  |
| --- | --- | --- | --- |
| 017 | Qofennan gakkiya yiirattu hayquwaa siyya eray? | A. Erayis B. Erikke |  |
| 018 | Yiira natta watti ichchisiyaakko eray | A. Erays  B. Erikke |  |
| 019 | Eriyaaba gidikko hagaappe kase oppe siyaddi? | A. Awa/aaye ayyeeppe  B. Payyatteta eranchatuppe  C. Laggetuppe |  |
| 020 | Ooppe ekko zoriyaa polliyakko dorayi | A. Awaa/aayee ayeeppe  B. Payyatteta eranchatuppe  C. Laggetuppe |  |
| 021 | Natta xiskkisanawu dorettiya baggay awugee? | A. Miyyiyan  B. Zokkuwaan  C. Ulluwan  D. Erikke |  |
| 022 | Natta xiskkisanawu injee heeray awee? | A. Awaanne aayeppe dumma kifiliyaa.  B. Awaaranne aayeera issi kifiliyan  C.Ta erikke |  |
| 023 | Yiiratta xiskkisanawu injee sohoy | A. Dumma indan barkka  B. Issi inddan yellidagetura  C.Erikke |  |
| 024 | Xisskissiyo wode Borkkota hittappe garsara wottiyogee nattussi injettes. | A. Injettes  B. Injettes |  |
| 025 | Natta suuree baggan xiskkissiyogee qoffenna yiirattu hayqquwaa teqqes | A. Ee  B. Teqqenna |  |
| 026 | Aayee xantta xalla xanttiyoyogaa giyoogee woygiyogee? | A. Usupun aginaa gakkanashin aayee xantta xalla xanttiyooga  B. Aayee xantta hara qummatura walakkiddi immiyooga |  |
| 027 | Ne yiiray buzzo inddan xisskki | A. ee  B.xiskkenna |  |
| 028 | Ne yiiray assi baynna kifiliyan barkka xiskki? | A. Ee  B. xiskkenna |  |
| 029 | Yiiray neennarakka giddin harara issippe xiskki | A. Ee  B.xiskkenna |  |
| 030 | Shaara wodiyan giddin yella simmada gayyiya/sijjara pugga eray | A. Ee  B. Erikke |  |
| 031 | Ne yiiray zokkuaappe hara baggan xiskki eri | A. Xiskkes  B. Xiskkenna |  |
| 032 | Ne yiira darotto awa baggan xisskissay? | A. Ulluwan  B. Zokkuwan  C. Miyyiyan |  |
