# Supplementary material for: Maternal knowledge and practice of safe infant sleep position in South Ethiopia: Implications for preventing sleep-related infant deaths
Source: PLoS One. 2026 Feb 3;21(2):e0339408. doi: 10.1371/journal.pone.0339408 (PMC12867215; doi:10.1371/journal.pone.0339408)
Supplement: Attachment — Submitted filename: Review report SISP.docx [file pone.0339408.s004.docx]

I appreciate the review invitation from the editor. I am also grateful to the authors for their efforts. I remarked on comments and suggestions that I thought could improve the integrity and readability of the author's work. Please find them below.

Title:

- Please, make the title more informative.

Introduction

- This section lack coherence and logical organization of sentences and paragraphs. I suggest the authors to rewrite the introduction.
- The authors did not cite source appropriately. They would cite sources immediately after each sentences rather than citing all sources together at the end paragraphs. In addition, the citation in a sentence “Placing infants on their backs to sleep has become the recommended sleep position by major health organizations, including the AAP, the Centers for Disease Control and Prevention (CDC), and the World Health Organization (WHO)[7, 8].” seems incomplete.
- Authors did not show the magnitude and severity of the problem. For instance, they simply report the occurrence high infant mortality. They would show how many of infant mortality are accounted to unsafe sleeping position, including SIDS.
- There are also nonspecific paragraphs.

Method

- Would not you think that the exclusion of “Infants presented to clinic by non-caregivers/not upbringing the infant. And Infants whose mothers are health care workers.” cause sampling bias?
- “Three hospitals were selected by random sampling method, and then proportional allocation was done for each hospital in terms of the previous year the same monthly report number of child care service to data collection period” Was it the previous year the same monthly report of the same age group service or general childcare?
- As you executed proportional allocation, you would put the Kth value used for each hospital.
- Is the tool self-developed or adapted from previous literature? Is it validated?
- At which hospital unit you contacted the mothers of infants? How did you managed the emotions of mothers of severely sick infants? How many minutes did the interview took?
- “Respondents who answer greater than the mean score of questions provide for them regarding knowledge will be considered as having good knowledge. While those answers below the mean score are those with poor knowledge.” This sentence is duplicated.
- How did you score Knowledge about SIDS?
- How did you manage the consent signing process for undedicated mothers?

Results

- What is your reference for covariates?
- Put the income category of the international currency ($) corresponding to that of the local during the study period too.
- I doubt the credibility of your data. Participants who attended higher education outweigh the other categories. This finding is contrary to national data (<https://www.weforum.org/reports/ab6795a1-960c-42b2-b3d5-587eccda6023>). Would you justify it, please?
- Please, convert interrogatives in the tables to variable form. Avoid the terms “you” and “your”.
- “While the other 326 (57.3%) of respondents reported that they had not practiced the safe infant sleep position.” This statement is not necessary.
- It’s better to minimize the decimals of AOR to 2 digits or follow the journal’s guideline.
- I suggest removing the p-value of the COR or using an asterisk instead. It congests the table.

Discussion:

- “The present study is distinguished by its notable strengths, primarily pertaining to the utilization of a large sample size and the establishment of diverse settings.” As you have used a logistic regression analysis to test multiple variables, I think this is not convincing elaboration. Read the rule of thumb for sample size in logistic regression” for your reference.
- Conclusion:
- The recommendation section is duplicated.

General suggestions:

Please, correct grammar, punctuation, and tense errors.
