# Supplementary material for: Maternal knowledge and practice of safe infant sleep position in South Ethiopia: Implications for preventing sleep-related infant deaths
Source: PLoS One. 2026 Feb 3;21(2):e0339408. doi: 10.1371/journal.pone.0339408 (PMC12867215; doi:10.1371/journal.pone.0339408)
Supplement: Attachment — Submitted filename: Infant safe sleeping comment.docx [file pone.0339408.s005.docx]

**General Comments:**
The study addresses an important public health issue—safe infant sleep practices—in a region where such data are scarce. The topic is highly relevant, given the global emphasis on reducing Sudden Unexpected Infant Death (SUID) and the lack of localized evidence in Ethiopia. The manuscript is well-structured, but some areas require clarification, refinement, or correction to enhance its quality and impact.

**Strengths:**

1. **Relevance and Importance:**
   - The study fills a critical gap in understanding safe sleep practices among mothers in South Ethiopia, contributing to global efforts to reduce infant mortality linked to unsafe sleep environments.
   - The focus on sociodemographic and knowledge-based determinants provides actionable insights for targeted interventions.
2. **Methodology:**
   - The use of a cross-sectional design with a large sample size (569 participants) is appropriate for assessing prevalence and associations.
   - Systematic sampling and rigorous data quality controls (e.g., pre-testing, Cronbach’s alpha for reliability) strengthen the validity of the findings.
   - Multivariable logistic regression was correctly employed to identify significant factors associated with safe sleep practices.
3. **Results and Discussion:**
   - The prevalence of safe sleep practices (42.7%) is clearly reported, with comparisons to other studies to contextualize the findings.
   - The discussion effectively interprets the results, highlighting discrepancies with prior research and plausible explanations (e.g., cultural, socioeconomic, and healthcare access differences).
4. **Ethical Considerations:**
   - Ethical approval and informed consent procedures are clearly documented, adhering to best practices for human subject research.

**Areas for Improvement:**

**1. Language and Grammar:**

- The manuscript contains minor grammatical errors and awkward phrasing. Examples:
  - "The unsafe position of the child while sleeping is associated with sudden and unexpected death in infancy." → *"Unsafe infant sleep positions are associated with sudden unexpected infant death (SUID)."*
  - "Efforts should be made to make accurate information accessible..." → *"Efforts should focus on improving access to accurate information..."*
- **Recommendation:** A thorough proofreading by a native English speaker or professional editing service would enhance readability.

**2. Clarity and Flow:**

- Some sections, such as the sampling procedure, are overly technical and could be simplified for broader readability.
- The abstract’s conclusion ("Efforts should be made to make accurate information accessible...") is vague. Specify *how* these efforts should be implemented (e.g., via antenatal education, community campaigns).

**3. Data Analysis:**

- The justification for using a *p*-value threshold of <0.25 in bivariate analysis (to select variables for multivariable regression) is unconventional. While this approach reduces the risk of excluding potentially significant variables, it should be explicitly justified in the methods section.
- The multicollinearity check (VIF < 2.9) is noted, but a brief discussion of how this was addressed (e.g., variable exclusion) would strengthen the analysis transparency.

**4. References:**

- The references are comprehensive and relevant, but some citations lack proper formatting (e.g., inconsistent use of italics for journal names, missing DOI/URLs).
- **Example:** "Pediatrics, 2022. **150**(1): p. e2022057991." → *Pediatrics*. 2022;150(1):e2022057991. doi: [insert DOI].*
- **Recommendation:** Ensure all references adhere to PLOS ONE’s style guidelines.

**5. Limitations:**

- The limitation regarding the lack of qualitative data (e.g., mothers’ rationale for sleep positions) is valid. However, the authors could propose future research directions to address this gap.
- The cross-sectional design limits causal inference; this should be explicitly noted in the limitations section.

**6. Recommendations:**

- The recommendations for policymakers and health professionals are practical but could be more specific. For example:
  - *"Hospitals should integrate safe sleep education into antenatal and postnatal care protocols, using visual aids (e.g., posters, videos) to demonstrate proper sleep positioning."*
  - *"Community health workers could conduct home visits to reinforce safe sleep practices, particularly in rural areas with limited healthcare access."*

**Overall Assessment:**

The manuscript presents valuable findings on a understudied topic in Ethiopia. With minor revisions to improve clarity, grammar, and methodological transparency, it would be suitable for publication in *PLOS ONE*. The study’s implications for reducing infant mortality align well with the journal’s focus on global health equity.

**Suggested Action:**

- **Accept with minor revisions**, addressing the above points.
- A thorough language edit and reference formatting check are strongly recommended.

**Additional Notes:**

- The figures and tables are clear and support the text effectively.
- The ethical and data availability statements comply with journal requirements.
