# Supplementary material for: Maternal knowledge and practice of safe infant sleep position in South Ethiopia: Implications for preventing sleep-related infant deaths
Source: PLoS One. 2026 Feb 3;21(2):e0339408. doi: 10.1371/journal.pone.0339408 (PMC12867215; doi:10.1371/journal.pone.0339408)
Supplement: Attachment — Submitted filename: Response to Reviewers.docx [file pone.0339408.s007.docx]

# Point-by-Point Response to Reviewers and Academic Editor

We would like to express our sincere gratitude to the Academic Editor and the reviewers for their valuable time, thoughtful comments, and constructive suggestions on our manuscript titled “Safe Sleep Practices for Infants: Prevalence and Determinants Among Mothers in South Ethiopia”

We have carefully reviewed each comment and made the necessary revisions to improve the clarity, coherence, and overall quality of the manuscript. In response to the feedback, we have reorganized key sections, clarified our methodology and justifications, strengthened the discussion with appropriate references, and improved the language throughout the manuscript.

We have prepared a detailed point-by-point response addressing all comments and clearly indicated the changes made in the revised version of the manuscript using track changes. We trust that the revisions have addressed the concerns raised and brought the manuscript to a standard suitable for publication in PLOS ONE.

We sincerely appreciate the opportunity to revise and resubmit our work and look forward to your favorable consideration.

Respectfully,
**Alemayehu B.Tekle (corresponding author)**
On behalf of all co-authors

**Manuscript Title:**
Safe Sleep Practices for Infants: Prevalence and Determinants Among Mothers in South Ethiopia

**Authors:**
Tamene D. Lachore, Tamirat T. Bekele, Alemayehu B. Tekle*, Nega Degefa, Genet Assefa

## Response to Reviewer 1

**Title**
1. Please make the title more informative.

**Response:** We have revised the title to: **“Maternal Knowledge and Practice of Safe Infant Sleep Position in South Ethiopia: Implications for Preventing Sleep-Related Infant Deaths’’** to reflect the study objectives and results more clearly.

**Introduction**
2. This section lack coherence and logical organization of sentences and paragraphs. I suggest the authors to rewrite the introduction.

**Response:** We have rewritten the Introduction for better logical flow, providing a clearer link between unsafe sleep practices and infant mortality.

3. *The authors did not cite source appropriately. They would cite sources immediately after each sentences rather than citing all sources together at the end paragraphs. In addition, the citation in a sentence “Placing infants on their backs to sleep has become the recommended sleep position by major health organizations, including the AAP, the Centers for Disease Control and Prevention (CDC), and the World Health Organization (WHO)[7, 8].” seems incomplete.*

**Response:** We have adjusted the placement of citations, ensuring that each claim is immediately supported by a reference.

4. *Authors did not show the magnitude and severity of the problem. For instance, they simply report the occurrence high infant mortality. They would show how many of infant mortality are accounted to unsafe sleeping position, including SIDS*

**Response:** We have included statistics showing SIDS-related deaths globally, in the U.S., UK, Italy, and Africa. We now clearly show how unsafe sleep practices contribute to SUID and SIDS.

**Methods**
5. *Would not you think that the exclusion of “Infants presented to clinic by non-caregivers/not upbringing the infant. And Infants whose mothers are health care workers.” cause sampling bias?*

**Response:** We have now acknowledged the potential for sampling bias due to these exclusions in the Limitations section.

6. *“Three hospitals were selected by random sampling method, and then proportional allocation was done for each hospital in terms of the previous year the same monthly report number of child care service to data collection period” Was it the previous year the same monthly report of the same age group service or general childcare?*

**Response:** We clarified that proportional allocation was based on the number of child health service visits for infants (<1 year) reported during the same months of the previous year.

7. ***As you executed proportional allocation, you would put the Kth value used for each hospital.***

**Response:** We have added the K-values calculated for each hospital using systematic sampling.

8. ***Is the tool self-developed or adapted from previous literature? Is it validated?***

**Response:** The questionnaire was adapted from published literature and theoretical framework.. Pre-testing and Cronbach’s alpha (0.741) confirmed its reliability. We updated the Methods section accordingly.

9. At which hospital unit were mothers interviewed? How were mothers of sick infants handled? How many minutes did the interview took?

**Response:** We now specify that interviews were conducted in the Child Health Clinics after services were provided. Mothers of severely sick infants were excluded to minimize distress. Each interview took approximately 15–20 minutes.

10. *“Respondents who answer greater than the mean score of questions provide for them regarding knowledge will be considered as having good knowledge. While those answers below the mean score are those with poor knowledge.” This sentence is duplicated.*

**Response: We corrected this redundancy by retaining only one clear definition.**

**11 *How did you score Knowledge about SIDS?***

**Response: We appreciate the reviewer’s attention to detail. However, assessing maternal knowledge specifically about SIDS was not among the primary objectives of this study. The focus of the knowledge assessment was on safe infant sleep position practices. While some questions in the tool indirectly referenced aspects related to SIDS, they were not scored or analyzed separately. We have clarified this in the methods section to avoid confusion.**
12. *How did you manage the consent signing process for uneducated mothers?*

**Response:** For illiterate mothers, verbal consent was obtained and thumbprints were used if needed. We added this information to the Ethical Considerations.

**Results**
13. *What is your reference for covariates?*

**Response:** Covariates were chosen based on published literature and theoretical framework

14. *Put the income category of the international currency ($) corresponding to that of the local during the study period too.*

**Response:** Income was converted using the 2024 exchange rate (1 USD ≈ 55 ETB) and indicated in footnotes.

15. *I doubt the credibility of your data. Participants who attended higher education outweigh the other categories. This finding is contrary to national data (<https://www.weforum.org/reports/ab6795a1-960c-42b2-b3d5-587eccda6023>). Would you justify it, please?*

**Response:** Because the study sampled mothers attending urban and hospital-based services, education levels were expected to be higher. This was added to the Limitations.

16. *Please, convert interrogatives in the tables to variable form. Avoid the terms “you” and “your”.*

**Response:** We have revised all tables to use neutral variable names.

17. *“While the other 326 (57.3%) of respondents reported that they had not practiced the safe infant sleep position.” This statement is not necessary.*

**Response:** We have removed the redundant sentence from Results.

18. *It’s better to minimize the decimals of AOR to 2 digits or follow the journal’s guideline.*

**Response:** All odds ratios are now rounded to two decimal places for clarity.

19. *I suggest removing the p-value of the COR or using an asterisk instead. It congests the table.*

**Response:** We have removed p-values for COR to decongest the tables.

20. ***“The present study is distinguished by its notable strengths, primarily pertaining to the utilization of a large sample size and the establishment of diverse settings.” As you have used a logistic regression analysis to test multiple variables, I think this is not convincing elaboration. Read the rule of thumb for sample size in logistic regression” for your reference.***

**Response:** We corrected the explanation by referencing the rule of thumb for logistic regression.

21. The Recommendation section is duplicated.

**Response:** We have merged and streamlined the Conclusion and Recommendations.

22. Correct grammatical errors.

**Response:** The manuscript underwent a detailed grammar and language review.

## Response to Reviewer 2

## 1. *The manuscript contains minor grammatical errors and awkward phrasing.*

**Response:** The manuscript was thoroughly proofread, and sentences were rephrased for clarity.

2. *Some sections, such as the sampling procedure, are overly technical and could be simplified for broader readability.*

**Response:** We simplified the explanation of the sampling technique and methods to make it more readable.

3. *The abstract’s conclusion ("Efforts should be made to make accurate information accessible...") is vague. Specify how these efforts should be implemented (e.g., via antenatal education, community campaigns).*

**Response:** We specified that efforts should include integrating education into antenatal and postnatal services.

4. Unconventional threshold for candidate variables.The justification for using a *p*-value threshold of <0.25 in bivariate analysis (to select variables for multivariable regression) is unconventional. While this approach reduces the risk of excluding potentially significant variables, it should be explicitly justified in the methods section.

**Response:** We justified the choice of p < 0.25 as a method to avoid excluding important variables, citing regression best practices.

5. Discuss how multicollinearity was checked.The multicollinearity check (VIF < 2.9) is noted, but a brief discussion of how this was addressed (e.g., variable exclusion) would strengthen the analysis transparency.

**Response:** Multicollinearity was checked using VIF; all values were <3.0. We included this explanation.

6. *The references are comprehensive and relevant, but some citations lack proper formatting (e.g., inconsistent use of italics for journal names, missing DOI/URLs).*

**Response:** References were reformatted per PLOS ONE guidelines and updated with DOIs where available.

7. *The limitation regarding the lack of qualitative data (e.g., mothers’ rationale for sleep positions) is valid. However, the authors could propose future research directions to address this gap.*

**Response:** We acknowledged this in the Limitations section and suggested future longitudinal studies.

8. *The cross-sectional design limits causal inference; this should be explicitly noted in the limitations section.*

**Response:** We acknowledged this in the Limitations section.

9. *The recommendations for policymakers and health professionals are practical but could be more specific.*

**Response:** We provided detailed recommendations for health professionals, hospitals, and policymakers.

## Response to Academic Editor

1. *Some of your discussions are ambitious; as such, they are not sufficiently supported by research or study across the globe. Again, the justification in the discussion (why does your study vary or be consistent with other similar studies?) is also not supported by the study.*
   **Response:** We revised overambitious statements and ensured all interpretations were supported by evidence.
2. *There are editorial problems throughout the manuscript.*

**Response:** The manuscript was professionally edited for English grammar, punctuation, and style.

3. *After mentioning the full form for any phrase the first time, then you should use the abbreviated one in other parts of your manuscript.*

**Response:** We corrected all abbreviation usage throughout the text.

4. ***Finally, please check all references to ensure that none of the cited articles have been retracted. You can use the Retraction Watch database, available here (http://retractiondatabase.org/)***

****Response**:** We checked all references via Retraction Watch Data Base (RWDB) and confirmed that none have been retracted.
