# Supplementary material for: Maternal knowledge and practice of safe infant sleep position in South Ethiopia: Implications for preventing sleep-related infant deaths
Source: PLoS One. 2026 Feb 3;21(2):e0339408. doi: 10.1371/journal.pone.0339408 (PMC12867215; doi:10.1371/journal.pone.0339408)
Supplement: Attachment — Submitted filename: Point by point response.docx [file pone.0339408.s008.docx]

# Point-by-Point Response to Reviewers and Academic Editor

**Dr. Ilker Kacer, Assoc. Prof. M.D.**
Academic Editor, PLOS ONE
[em.pone.0.95206d.724294ad@editorialmanager.com]

**RE:** Revised Submission – Manuscript ID: PONE-D-25-10950R1
**Title:** Maternal Knowledge and Practice of Safe Infant Sleep Position in South Ethiopia: Implications for Preventing Sleep-Related Infant Deaths

Dear Dr. Kacer,

We sincerely thank you and the reviewers for your thoughtful and constructive comments on our manuscript. We have carefully revised the manuscript in accordance with the feedback provided and believe that the revised version is substantially improved.

We would also like to respectfully bring to your attention that **all comments provided by Reviewer #5** appear to relate to a different manuscript, focusing on fecal-oral parasitic transmission in public toilets. As these comments are not applicable to our study on safe infant sleep practices, we have noted this in our response letter. However, if any part of Reviewer #5’s feedback was intended for our submission, we are happy to provide clarification or additional revisions as needed.

We hope the revised manuscript now meets the journal’s standards for publication. Thank you again for the opportunity to revise and resubmit our work to PLOS ONE. We greatly appreciate your time and consideration.

Sincerely,
**Dr. Alemayehu B. Tekle, MD, ECCM**
Corresponding Author
School of Medicine, Wolaita Sodo University, Ethiopia
Email: [alemayehub.tekle@wsu.edu.et](mailto:alemayehub.tekle@wsu.edu.et)

### ****Reviewer #3****

***Comment 1:*** *There are some grammatical errors in the paper, e.g., “Mother in low” instead of “Mother-in-law.”*

**Response:** Thank you for pointing this out. We have corrected the phrase “Mother in low” to “Mother-in-law” and thoroughly reviewed the manuscript to fix additional typographical and grammatical errors.

***Comment 2:*** *Some of the terms were not clearly defined, including the 10 knowledge assessment items. The actual list should be provided and clearly defined.*

**Response:** We appreciate this suggestion. We have now included a description of the 10 knowledge assessment items in the **Operational Definitions** section. These items are derived from established literature and assess maternal understanding of sleep position safety. The questionnaire is also provided as Supporting Information.

***Comment 3:*** *Ensure all figures, e.g., Fig 2 and Fig 3, are embedded with clear titles and legends. Currently, they are referenced but seem separate.*

**Response:** Thank you for this helpful comment. We have now ensured that **all figures (including Figure 2 and Figure 3)** are embedded in the manuscript with **clear and descriptive titles and legends**. While the figures will also be submitted separately in accordance with journal guidelines, we have revised the manuscript to improve clarity and ease of interpretation.

### ****Reviewer #4****

***Comment 1:*** *A systematic sampling method was used, but exclusions (e.g., healthcare worker mothers) may introduce bias. Please justify.*

**Response:** Thank you for your observation. We have added justification for the exclusion of healthcare worker mothers. Mothers who are healthcare professionals were excluded to avoid potential bias due to their advanced medical knowledge, which may not reflect the general population's awareness and behavior.

***Comment 2:*** *The informed consent process for illiterate participants needs more detail.*

**Response:** We agree. We have revised the **Ethical Consideration** section to describe how verbal consent and thumbprints were used for illiterate participants, ensuring ethical compliance and participant autonomy.

***Comment 3:*** *The manuscript mentions checking multicollinearity using VIF but doesn’t explain how it was addressed.*

**Response:** Thank you. We now state that multicollinearity was assessed using VIF, and no significant multicollinearity was detected (maximum VIF = 2.9). This is now clarified in the **Data Analysis** section.

### ****Reviewer #5****

**General Comments:**

We sincerely appreciate Reviewer #5’s time and effort in providing feedback. However, upon careful review, we believe that all of the comments provided (e.g., references to fecal-oral parasitic transmission, public toilets, and related keywords such as Giardia lamblia) pertain to a **different manuscript** and are not relevant to our study, which focuses on **maternal knowledge and practice of safe infant sleep position in South Ethiopia**.

Should any of these comments have been intended for our manuscript, we would be grateful for clarification and are happy to respond accordingly.
