# Supplementary material for: Maternal knowledge and practice of safe infant sleep position in South Ethiopia: Implications for preventing sleep-related infant deaths
Source: PLoS One. 2026 Feb 3;21(2):e0339408. doi: 10.1371/journal.pone.0339408 (PMC12867215; doi:10.1371/journal.pone.0339408)
Supplement: Attachment — Submitted filename: Response to Reviewer Comments.docx [file pone.0339408.s009.docx]

Point-by-Point Response to Reviewers and Academic Editor

Dr. Ilker Kacer, Assoc. Prof. M.D.

Academic Editor, PLOS ONE

[em.pone.0.969c88.cc76924f@editorialmanager.com]

RE: Revised Submission – Manuscript ID: PONE-D-25-10950R2

Title: Maternal Knowledge and Practice of Safe Infant Sleep Position in South Ethiopia: Implications for Preventing Sleep-Related Infant Deaths

Dear Dr. Kacer,

We are pleased to resubmit our revised manuscript entitled:
“Maternal Knowledge and Practice of Safe Infant Sleep Position in South Ethiopia: Implications for Preventing Sleep-Related Infant Deaths.”

We sincerely thank you and the reviewers for the constructive and insightful comments provided on our initial submission. We carefully considered all suggestions and have revised the manuscript accordingly to improve clarity, methodological transparency, and overall scientific quality.

We have included a detailed, point-by-point response document addressing every reviewer comment.

We believe that these revisions have substantially strengthened the manuscript, and we are hopeful that the revised manuscript will now be suitable for publication in PLOS ONE.

Sincerely,

Dr. Alemayehu B. Tekle, MD, ECCM

Corresponding Author

School of Medicine, Wolaita Sodo University, Ethiopia

Email: [alemayehub.tekle@wsu.edu.et](mailto:alemayehub.tekle@wsu.edu.et)

### ****Comment 1:****

Abstract: not sure if there is a word limit, but could be shortened a bit if needed (especially Methods section). Background: should “sudden and unexpected death in infancy” be “sudden unexpected infant death” or “sudden infant death syndrome”? Would try to keep terms consistent for clarity if possible.

**Response:**
Thank you for the suggestion. We have reviewed the abstract and shortened the Methods section to enhance clarity and conciseness while maintaining essential details.
We have also standardized terminology throughout the abstract and manuscript by replacing “sudden and unexpected death in infancy” with **“sudden unexpected infant death (SUID)”** and used **“sudden infant death syndrome (SIDS)”** only when specifically referring to that subset of unexplained cases.

### ****Comment 2:****

Introduction (page 2): first full paragraph (“Each year infants die…”) and second full paragraph (“An unsafe sleeping position…”) should probably be combined for clarity. For this audience, may want to distinguish the term SUDI from SIDS since they are being used together in this paragraph and may be confusing. “SIDS is nearly 2,500 in the United States”: is this deaths per year? Please clarify.

**Response:**
We appreciate this clarification. We have combined the two paragraphs together. SIDS was used throughout manuscript. SIDS is nearly 2,500 deaths per year in the United States.

### ****Comment 3:****

Introduction (page 3): Paragraph that starts with “Safe sleep position practice…” seems out of place since the discussion has shifted from SIDS recommendations to the experience in Africa. Would suggest moving earlier in the Introduction.

**Response:**
Thank you. We have moved this paragraph to follow the background section on SIDS recommendations, so that the transition from international evidence to the African context is more logical.

### ****Comment 4:****

Exclusion criteria (page 6): “Infants presented to…”: seems redundant since mother is needed for inclusion. “Congenital diseases”: maybe change to "conditions" and would clarify that these would potentially result in children being recommended to sleep in a different position.

**Response:**
We agree. The redundant phrase was deleted. “Congenital diseases” was replaced with **“congenital conditions that may require alternative sleeping positions”** to clarify the rationale for exclusion.

### ****Comment 5:****

Operational Definition and Measurement (page 11): “Respondents who scored the mean and above”: Would clarify if this was the mean from this cohort or a pre-set score (i.e., was the design so that exactly half would be scored as having "good knowledge?")

**Response:**
Thank you for highlighting this important point. In our original analysis, we used a pre-set cut-off score of 50% (≥5 out of 10 correct responses) to categorize mothers as having “good knowledge.” This approach is consistent with similar studies conducted in Ethiopia and other LMIC settings.

However, we unintentionally described this as “mean and above” in the operational definition section, which was a wording error.

To correct this and ensure clarity and consistency, we have revised the operational definition in the Methods section to reflect the actual pre-set scoring system used in the analysis. This clarification does not affect the results or interpretation of the study.

### ****Comment 6:****

Sociodemographic characteristics of the respondents (page 14): is “housewives” the preferred term locally? Are they not currently working because they have an infant at home?

**Response:**
Yes, “housewife” is a commonly used and culturally accepted term in the Ethiopian context and we have clarified in the text that many mothers are temporarily not working due to infant care responsibilities.

### ****Comment 7:****

Family income (Table 2): is this per week/month?

**Response:**
Revised to read **“Monthly family income”** in both the text and table footnote for clarity.

### ****Comment 8:****

Page 18 (paragraph at bottom of page): “Almost all infants 98.6% were sleep other than supine”: This sentence is confusing and is key to the manuscript. Would revise to clarify. Based on the table, seems like this is a combination of infants who primarily sleep on their back and maybe those who are able to roll onto their back from other positions?

**Response:**
We agree this needed clarification. The sentence now reads:

“Almost all infants (98.6%) were placed to sleep in positions other than supine. Among them, 56.1% usually slept on their side, and 1.2% in the prone position, while only 42.7% were consistently placed on their backs.”

### ****Comment 9:****

Factors associated with safe infant sleep position practice (page 21): “family income parity”: Does "parity" here refer to family income or the number of babies a mother has had? Please clarify.

**Response:**
Thank you for catching this. The phrase “family income parity” was an editing oversight. It was the typing error and corrected. Parity here refers to the number of babies a mother has had.

### ****Comment 10:****

Material occupation (Figure 6, page 22): would change p=0.000 to p<0.001 or similar.

**Response:**
Revised as suggested. All “p = 0.000” values have been replaced with **“p < 0.001.”**

### ****Comment 11:****

Discussion (second paragraph, page 25): “The prevalence found in this study was lower…”: Was this asked in the same way ("how does the infant usually sleep?") in this cohort compared with those other studies? Older infants, even if initially placed in the supine position, may roll over, and not sure if this is part of the reason that number could be lower.

**Response:**
Thank you for the thourough observation. Yes these differences may be due to variations in methodology, study settings, socioeconomic status, and public health infrastructure as stateded on the discussion. And yes we agree different phrasing might contribute to the lower prevalence. So we have added the following sentence in the Discussion to add some light to the reader.:

“The observed lower prevalence may partly be due to the phrasing of the question (‘how does the infant usually sleep?’), which reflects both maternal placement and infant movement during sleep. As infants grow older, spontaneous rolling may reduce the likelihood of maintaining a supine position despite being initially placed on their backs.”

### ****Comment 12:****

General: “data was” should be changed to “data were” through the manuscript.

**Response:**
All instances of “data was” have been systematically corrected to **“data were.”**
